# The Utility of Laboratory Parameters for Cardiac Inflammation in Heart Failure Patients Hospitalized with SARS-CoV-2 Infection

**DOI:** 10.3390/diagnostics12040824

**Published:** 2022-03-27

**Authors:** Ciprian Nicolae Pilut, Cosmin Citu, Florin Gorun, Felix Bratosin, Oana Maria Gorun, Bogdan Burlea, Ioana Mihaela Citu, Mirela Loredana Grigoras, Diana Manolescu, Adrian Gluhovschi

**Affiliations:** 1 Department of Microbiology, Faculty of Medicine, “Victor Babes” University of Medicine and Pharmacy, Eftimie Murgu Square 2, 300041 Timisoara, Romania; pilut.ciprian@umft.ro; 2 Department of Obstetrics and Gynecology, “Victor Babes” University of Medicine and Pharmacy Timisoara, Eftimie Murgu Square 2, 300041 Timisoara, Romania; gorun.florin@umft.ro (F.G.); adigluhovschi@yahoo.com (A.G.); 3 Methodological and Infectious Diseases Research Center, Department of Infectious Diseases, “Victor Babes” University of Medicine and Pharmacy, 300041 Timisoara, Romania; felix.bratosin7@gmail.com; 4 Department of Obstetrics and Gynecology, Municipal Emergency Clinical Hospital Timisoara, 1-3 Alexandru Odobescu Street, 300202 Timisoara, Romania; oanabalan@hotmail.com (O.M.G.); bogdanburlea@yahoo.com (B.B.); 5 Department of Internal Medicine I, “Victor Babes” University of Medicine and Pharmacy Timisoara, Eftimie Murgu Square 2, 300041 Timisoara, Romania; citu.ioana@umft.ro; 6 Department of Anatomy and Embryology, “Victor Babes” University of Medicine and Pharmacy Timisoara, Eftimie Murgu Square 2, 300041 Timisoara, Romania; grigoras.mirela@umft.ro; 7 Department of Radiology, “Victor Babes” University of Medicine and Pharmacy Timisoara, Eftimie Murgu Square 2, 300041 Timisoara, Romania; dmanolescu@umft.ro

**Keywords:** SARS-CoV-2, COVID-19, heart failure, mortality risk, inflammatory markers

## Abstract

COVID-19 has been associated with cardiovascular consequences, including myocardial infarction, thromboembolic events, arrhythmia, and heart failure. Numerous overlapping mechanisms, such as the IL-6 dependent cytokine storm and unopposed angiotensin II stimulation, could be responsible for these consequences. Cardiac damage is hypothesized to be a consequence of the direct viral infection of cardiomyocytes, resulting in increased metabolic demand, immunological activation, and microvascular dysfunction. Patients with pre-existing chronic heart failure are therefore at increased risk of decompensation, further heart damage, and significant health deterioration. Based on the aforementioned assumptions, we developed a study aiming to provide a detailed description of changes in biological parameters and cardiac injury markers of patients with heart failure and SARS-CoV-2 infection by correlating them with the clinical presentation and COVID-19 vaccination status, to predict the probability of ICU admission based on their initial hospital presentation. A two-year retrospective study was performed on heart failure patients with a history of SARS-CoV-2 infection and detailed records of biological biomarkers; a total of 124 eligible patients with COVID-19 and 236 without COVID-19 were recruited. Patients with heart failure and SARS-CoV-2 infection had significantly elevated baseline biological parameters and cardiac markers compared to those without COVID-19. Several cardiac injury markers were identified as significant independent risk factors for ICU admission: CK-MB (HR = 4.1, CI [2.2–6.9]), myoglobin (HR = 5.0, CI [2.3–7.8]), troponin-I (HR = 7.1 [4.4–9.6]), troponin-T (HR = 4.9, CI [1.7–7.4]). The elevation of a basic panel of acute inflammation markers (CRP, IL-6, fibrinogen), D-dimers, and BNP was also a significant risk factor. The follow-up of survivors at four weeks after viral clearance determined a worsened clinical picture by NYHA classification, worsened cardiac ultrasound findings, and a mild improvement in cardiac and inflammatory markers. Increased levels of myocardial damage parameters in association with cardiac ultrasound findings and basic inflammatory markers may enable early risk assessment and triage in hospitalized heart failure patients infected with SARS-CoV-2.

## 1. Introduction

The coronavirus disease 2019 (COVID-19) has the potential to induce viral pneumonia and other cardiovascular problems. In early investigations, more than 30% of patients hospitalized with COVID-19 had underlying comorbidities, including hypertension, cardiovascular disease, and other cardiovascular risk factors such as diabetes mellitus [1]. Cardiovascular disease prevalence varied significantly according to study size for COVID-19 populations, ranging from 40% in small samples of COVID-19 patients [2] to as low as 2–4% in large studies of more than a thousand COVID-19 patients [3]. As the pandemic continued, a meta-analysis of COVID-19 studies determined that the prevalence of hypertension, cardiovascular and cerebrovascular illness and diabetes was between 15% and 20% [4]. Additionally, it is known that male sex, advanced age, and the existence of hypertension, diabetes mellitus, cardiovascular illnesses, and cerebrovascular disorders, as well as consequences of acute cardiac injury, cardiomyopathy, and heart failure, all contribute to death in COVID-19 patients. In a large series of more than 40 thousand COVID-19 patients, individuals with coronary heart disease or heart failure had a 10% higher fatality rate than the general mortality rate of 2% [5]. Coronary heart disease and myocardial damage coexisting were related to the greatest death rates among chronic conditions [6].

Heart failure was reported in over 20% of severe acute respiratory syndrome coronavirus 2 (SARS-Cov-2)-positive patients hospitalized with severe symptoms [7,8], and about half of those who died had heart failure as a consequence [9]. Additionally, individuals with underlying cardiovascular comorbidities, such as persistent hypertension, had a greater risk of developing heart failure [10]. Patients with heart failure have a significantly increased risk of thromboembolic events, acute respiratory distress syndrome (ARDS), severe hypotension, and mortality [11,12]. SARS-CoV-2-positive individuals who develop acute cardiac failure have a death rate of over 50% in these instances [13]. Cardiovascular signs and symptoms, such as dyspnea and chest discomfort, may have a high degree of overlap with COVID-19 [14,15]. Furthermore, cardiovascular problems have been seen to develop regularly during the disease’s course, and they should always be carefully monitored in severe COVID-19 necessitating hospitalization. These individuals were shown to be more likely to need hospitalization, and intensive care unit admission, and had higher death rates [16]. The wide variety and frequent occurrence of cardiovascular consequences in a respiratory illness such as COVID-19, as well as the predominance of individuals with cardiovascular comorbidities, indicate the disease’s complexity.

SARS-CoV-2 enters the host cell through the angiotensin-converting enzyme 2 (ACE2) [17]. Heart failure has an underlying alteration in the renin-angiotensin system (RAS)-, and the use of RAS inhibitors raises the ACE2 levels, which may facilitate SARS-CoV-2 damage to the lungs and heart [18]. As a result, individuals with coexisting cardiovascular disorders may directly increase the infection’s effect and severity. Moreover, the cytokine storm described in multiple cases of patients with COVID-19 mimics the clinical picture of multiple organ failure, including heart failure, which can further decompensate. Typically, the cytokine storm appears as an influenza-like symptom that may progress or become complex as a result of multi-organ damage [19]. Fever is usually persistent, with the most severe instances resulting in a very high body temperature [20]. Additionally, tiredness, headache, diarrhea, lymphadenopathy, hepatosplenomegaly, sensory alterations, and skin rash are typical signs [21]. Often, tachypnea and hypoxia are present, which might progress to ARDS. Severe infections may potentially cause acute renal injury, liver damage, and stress-related cardiomyopathy even in healthy individuals [22,23,24].

Therefore, the study attempted to provide a detailed description of serum findings and cardiac ultrasound findings of heart failure patients admitted to hospital for SARS-CoV-2 infection while aiming to correlate patients’ biological parameters with their clinical profile and determine the likelihood of intensive care unit (ICU) admission based on their initial hospital presentation.

## 2. Materials and Methods

### 2.1. Design and Ethics

This current single-center research study followed a retrospective cohort design of heart failure patients hospitalized for SARS-CoV-2 infection. The research was conducted at a tertiary hospital in western Romania, where patients were hospitalized in the COVID-19 unit of the Timisoara Municipal Emergency Hospital’s Internal Medicine Department between February 2020 and February 2022. The Ethics Committee of the “Victor Babes” University of Medicine and Pharmacy in Timisoara, Romania, as well as the Ethics Committee of the Timisoara Municipal Hospital, accepted the study protocol.

### 2.2. Patient Population and Inclusion/Exclusion Criteria

The inclusion criteria were established for all patients over the age of 18 with a history of hospitalization in our department for SARS-CoV-2 infection, as determined by real-time polymerase chain reaction (RT-PCR). A history of heart failure prior to hospital admission for COVID-19, identified in the hospital database, was mandatory to be included in the current study. Heart failure was defined by systolic or diastolic dysfunction on cardiac ultrasound, and clinical signs and symptoms determined by systemic or pulmonary congestion. Exclusion criteria comprised inadequate patient profiles in terms of imaging examinations and laboratory data, as well as records that lacked patient permission. Additionally, the cases without a secondary outpatient evaluation at four weeks after SARS-CoV-2 clearance were excluded from the current analysis. Data collection was conducted by trained physicians who volunteered to participate in this research and confirmed the database information against existing patient paper records. The variables analyzed included the background characteristics of patients (age, gender, body mass index (BMI)), cardiovascular risk factors (smoking, arterial hypertension, diabetes mellitus, dyslipidemia), comorbidities at admission (cerebrovascular disease, chronic kidney disease, chronic obstructive pulmonary disease), cardiac ultrasound findings, New York Heart Association (NYHA) classification, the status of COVID-19 vaccination, oxygen supplementation, COVID-19 severity, the prevalence of clinical manifestations at admission (cough, fatigue, myalgia/arthralgia, anosmia/ageusia, fever, headache, nasal congestion, rhinorrhea, diarrhea), and disease outcomes (ICU admission and in-hospital mortality).

### 2.3. Laboratory Data and Clinical Follow up

All participants received an initial outpatient COVID-19 examination and were categorized as having mild, moderate, or severe SARS-CoV-2 infection. All patients with pulmonary lesions were categorized as mild (30% pulmonary damage), moderate (30–60% pulmonary damage), or severe (more than 60% of lung area affected) based on computed tomography (CT) data. A complete medical history, clinical examination, electrocardiogram (ECG), and transthoracic echocardiography were all part of the cardiologic assessment. Following a routine examination of cardiac morphology and function, we determined the left ventricular mass index (LVMI) to detect left ventricular hypertrophy, defined as LVMI > 115 g/m^2^ for males and 95 g/m^2^ for females; the left atrial volume index (LAVI), with values greater than 34 mL considered pathological; and the presence of pericardial effusion and thickened pericardium greater than or equal to 4 mm in thickness. The left ventricular function was evaluated from an apical 4-chamber view, with values less than 50% considered abnormal; the lateral mitral annular plane systolic excursion (MAPSE) was measured, with values less than 10 mm considered pathological; and the left ventricular global longitudinal strain (LV-GLS) was quantified as pathologic when higher than 18%. The evaluation of diastolic dysfunction was performed using pulsed Doppler imaging in the apical 4-chamber view by recording the mitral inflow at the annulus of the mitral valve, together with the peak early diastolic velocity (E), the late diastolic velocity (A), and the E/A ratio. The right ventricular function was assessed from a 4-chamber view by visualizing the entire right ventricle. This included measuring the tricuspid annular plane systolic excursion (TAPSE), with values greater than 17 mm defining right ventricular dysfunction (RVD). The estimated pulmonary artery systolic pressure (sPAP) was computed using the peak tricuspid regurgitation velocity (TRV), as detected by continuous-wave Doppler, and the right atrial pressure, as obtained by monitoring the inferior vena cava diameter and its respiratory fluctuations. An sPAP level of 35 mmHg or higher at rest indicated pulmonary hypertension, with severity ranging from mild (35–44 mmHg) to moderate (45–60 mmHg) and severe (>60 mmHg). All measurements were carried out in accordance with the instructions of the existing guidelines [25].

Laboratory data comprised the blood cell count (RBC), platelet count (PLT), white blood cell count (WBC), neutrophils, monocytes, eosinophils, lymphocytes, hemoglobin (Hb), hematocrit (Ht), mean corpuscular volume (MCV), fasting glucose, alanine aminotransferase (ALAT), aspartate aminotransferase (ASAT), alkaline phosphatase (ALP), serum albumin, total proteins, total bilirubin, gamma glutamate transpeptidase (GGT), lactate dehydrogenase (LDH), prothrombin time (PT), partial thromboplastin time (APTT), creatinine, blood urea nitrogen (BUN), urinary albumin, estimate glomerular filtration rate (eGFR), total cholesterol, triglycerides, VLDL-C, LDL-C, HDL-C, total lipid, procalcitonin, c-reactive protein (CRP), interleukin-6 (IL-6), tumor necrosis factor-alpha (TNF-α), erythrocyte sedimentation rate (ESR), fibrinogen, brain natriuretic peptide (BNP), creatine kinase (CK), myoglobin, troponin I, troponin T, and lactate dehydrogenase (LDH). All patients were re-evaluated at four weeks after SARS-CoV-2 infection clearance and hospital discharge. The outcomes evaluated at 4 weeks comprised a clinical assessment by NYHA classification, cardiac ultrasound, and inflammatory markers.

### 2.4. Statistical Analysis

IBM SPSS v.26 and MedCalc v.20 were used for statistical analysis. The absolute and relative frequencies of categorical variables were calculated and compared with the Chi-square and Fisher’s test. The Kruskal–Wallis test was performed to compare group differences in nonparametric data. A Shapiro–Wilk test determined if the variables were normally distributed. The variables determined to be significantly different between comparison groups were included in a Cox regression analysis adjusted for confounding variables, with results expressed as hazard ratio (HR) and confidence interval (CI). A significance level of 0.05 was chosen for the alpha value.

## 3. Results

### 3.1. Background Analysis

A total of 124 patients with heart failure and a history of SARS-CoV-2 infection satisfied the inclusion criteria. Table 1 presents the background characteristics of heart failure patients with SARS-CoV-2 infection compared with a group of 236 heart failure patients without COVID-19. There were statistically significant differences between the study groups by cardiovascular risk factors, including diabetes mellitus (33.9% in the COVID-19 group vs. 24.2% in the no COVID-19 group, *p*-value = 0.049) and dyslipidemia (31.5% in the COVID-19 group vs. 21.6% in the no COVID-19 group, *p*-value = 0.040). The study groups were homogenous in the prevalence of comorbid conditions, except malignancies, where 9.7% of heart failure patients with COVID-19 had cancer, compared with 3.8% of heart failure patients without COVID-19 (*p*-value = 0.024). Patient groups were also significantly different depending on their heart failure clinical symptomatology measured on the NYHA scale (*p*-value = 0.002). Lastly, an important divergence in the patient background was the status of COVID-19 vaccination, where only 38.7% in the SARS-CoV-2 infection group had a complete vaccination scheme, compared with 53.8% in those who did not become infected (*p*-value = 0.006).

### 3.2. Laboratory Analysis

The laboratory profile of heart failure patients with and without COVID-19, presented in Table 2, was significantly different depending on the number of white blood cells, neutrophils, and lymphocytes. The median value of white blood cells in the COVID-19 group was 13.1, compared with 4.7 in the non-COVID-19 group (*p*-value < 0.001). The neutrophil count was also significantly increased in COVID-19 patients (7.4 × 10^3^ vs. 4.9 × 10^3^, *p*-value < 0.001). A total of 65.3% of heart failure patients with COVID-19 had the lymphocyte count outside the normal range, compared with 36.9% of heart failure patients without COVID-19 (*p*-value < 0.001). Liver function tests were also more altered during SARS-CoV-2 infection, indicating signs of liver damage or liver failure, where fasting glucose (*p*-value = 0.029), ALT (*p*-value = 0.038), AST (*p*-value = 0.033), and PT (*p*-value < 0.001) had significantly higher values compared with the no-COVID-19 group. Kidney dysfunction was more prevalent in heart failure patients with COVID-19, since the laboratory tests showed an important deviation from the normal range Creatinine (*p*-value = 0.002), BUN (*p*-value = 0.009), and eGFR (*p*-value = 0.045) were significantly increased in heart failure patients with COVID-19.

The inflammatory markers, excepting procalcitonin and IFN-γ, were all statistically significantly further from the normal range, as described in Table 2. A total of 71.8% of heart failure patients in the COVID-19 group had CRP values outside the normal range, compared with only 23.3% in patients without COVID-19 (*p*-value < 0.001); IL-6 in the disease group had a median of 48 pg/mL, compared with 15 pg/mL in the control group (*p*-value < 0.001); 53.2% of heart failure patients with COVID-19 had TNF-α values outside the normal range, while it was 15.7% outside normality in the control group (*p*-value < 0.001). ESR (*p*-value < 0.001), fibrinogen (*p*-value = 0.003), and D-dimers (*p*-value < 0.001) were all statistically significantly more elevated than in the other group. Patients with heart failure and SARS-CoV-2 infection had not only increased serum inflammatory markers, but also elevated cardiac injury markers, including CK-MB (median value 33 U/L vs. 26 U/L, *p*-value = 0.015), myoglobin (median value 3.9 nmol/L vs. 3.5 nmol/L, *p*-value = 0.023), troponin I (median value 0.5 ng/mL vs. 0.3 ng/mL, *p*-value = 0.040), and troponin T (median value 16 ng/mL vs. 12 ng/mL, *p*-value = 0.037).

**Figure 1:**
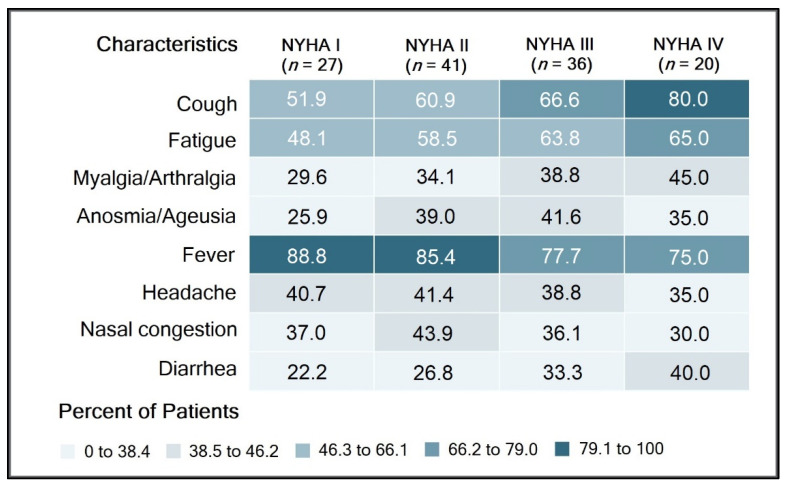
Comparison of signs and symptoms by NYHA classification of heart failure hospitalized patients with COVID-19.

The clinical analysis of heart failure patients with COVID-19 determined 27 (21.8%) patients classified as NYHA I, 41 (33.1%) patients with NYHA II, 36 (29.0%) patients with NYHA III, and 20 (16.1%) patients with NYHA IV. Most patients with NYHA IV (80.4%) complained of cough at admission, followed by fever (72.3%), and fatigue (67.1%). However, a higher proportion of patients with NYHA I and II heart failure presented with a fever (89.4% and 86.3%, respectively). Patients classified as NYHA IV had significantly more symptoms of myalgia/arthralgia and diarrhea than NYHA I patients (42.6% vs. 28.6%), respectively (39.2% vs. 22.6%), as seen in Figure 1.

A comparison of inflammatory markers by NYHA classification of hospitalized heart failure patients with COVID-19 identified statistically significant differences between the four groups. NYHA IV hospitalized patients with COVID-19 had the highest median values of fibrinogen (7.2 g/L, *p*-value = 0.048), BNP (1170 pg/mL, *p*-value < 0.001), CK-MB (37 U/L, *p*-value < 0.001) LDH (336/U/L, *p*-value = 0.012), myoglobin (3.9 nmol/L, *p*-value < 0.001), and troponins, as described in Table 3.

The cardiac marker comparison presented in Table 4 did not determine any significant differences between the groups of vaccinated and unvaccinated hospitalized heart failure patients with COVID-19. Although a higher proportion of patients in the unvaccinated group had the biological parameters outside the normal range, the median difference was not statistically significantly higher.

**Figure 2:**
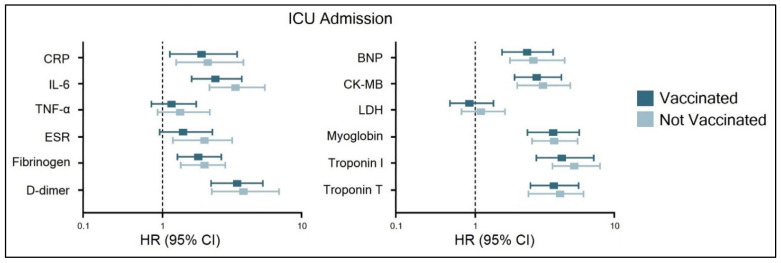
Risk factor analysis for ICU admission of heart failure hospitalized patients with COVID-19, stratified by status of COVID-19 vaccination.

The Cox regression analysis for ICU admission of heart failure hospitalized patients with COVID-19 stratified by status of complete COVID-19 vaccination scheme, presented in Figure 2, was adjusted for patient age and comorbidities. Significant independent risk factors were CRP, IL-6, fibrinogen, d-dimers, BNP, CK-MB, myoglobin, and troponins, where troponin I had the highest hazard ratio (HR) of 7.1 in unvaccinated patients, compared with an HR of 6.4 in those who were vaccinated. This was followed by D-dimers (HR = 5.6 in the vaccinated group, respectively HR = 6.1 in the unvaccinated group), and myoglobin (HR = 5.0 in the vaccinated group, respectively HR = 5.3 in the unvaccinated group). TNF-α, ESR, and LDH did not show a high risk for ICU admission.

### 3.3. Follow-Up

The follow-up at four weeks evaluated 96 patients out of 124 heart failure patients with COVID-19 who initially presented for the hospital admission. It was observed that a significantly higher proportion of patients (*p*-value = 0.047) had a worsened clinical picture of heart failure. From 20 (16.1%) patients with NYHA IV at admission, there were 29 (30.2%) NYHA IV at four weeks after viral clearance. Ultrasound examination at four weeks identified a statistically higher proportion of patients with pericardial effusion (18.5% vs. 32.3%, *p*-value = 0.018); a proportion of 59.4% patients with LV-GLS values outside normality (*p*-value = 0.036), 39.6% patients with LV-DD outside normal range (*p*-value = 0.041), and 33.3% patients with sPAP outside the normal range (*p*-value = 0.038). The inflammatory markers continued to be significantly elevated at the four-week evaluation in the analysis of procalcitonin, ESR, D-dimers, CK-MB, myoglobin, and troponins (Table 5).

## 4. Discussion

Our findings contribute significant evidence to the body of knowledge by confirming the value of cardiac damage indicators such as troponin, BNP, CK-MB, and myoglobin for assessing the likelihood of ICU admission and other potentially life-threatening complications or acute cardiac events. Additionally, a basic inflammatory panel was useful in estimating the probability of ICU admission during hospitalization for SARS-CoV-2 infection. This emphasizes the critical need for regularly assessing cardiac inflammatory markers and serum inflammatory parameters in these patients to aid in early predicting adverse outcomes and ICU admission. Thus, systematic evaluation of these biological markers may assist in risk stratification and illness classification and diagnosis in heart failure patients hospitalized for COVID-19.

Due to the increased risk of acute direct or indirect cardiac damage in COVID-19, cardiac troponin and other injury indicators are increased, which may imply a worse prognosis and death [26,27]. This discovery sparked considerable scholarly interest in the use of cardiac biomarkers and serum inflammatory markers as diagnostic and predictive techniques for cardiac problems associated with SARS-CoV-2 infection [28]. Cardiac troponin has previously been demonstrated to be a strong predictor of death, intensive care unit admission, and myocardial damage in individuals with COVID-19 [29]. This was confirmed in a meta-analysis of 10 studies involving nearly 4000 patients [30], in which higher troponin levels were associated with a significantly increased risk of intensive care unit admission, oxygen saturation of 90%, invasive mechanical ventilation, and in-hospital mortality (OR = 7.9), data that corroborate our findings. On the other hand, several studies have shown that troponin levels on the first day of admission had a strong negative predictive value for predicting death from any cause [31].

Echocardiography, as depicted in our study, may be critical in this context for the early detection of primary or COVID-19-induced myocardial injury. Still, biomarkers are even more critical in COVID-19 patients for diagnostic and prognostic reasons since they help avoid viral transmission during trials in which patients are evaluated using the same device. Although we evaluated a basic sample of parameters that are widely available and easy to determine, other studies identified more specific cardiac injury markers that are useful in predicting outcomes in SARS-CoV-2-infected patients. After COVID-19 infection, it was described by Izquierido et al. [32] that myocardial damage during hospitalization was associated with thicker myocardial walls and greater pulmonary artery pressure on six-month follow-up echocardiograms, which is consistent with other findings [33], as well as with our evaluation at four weeks. The same authors analyzed the serum levels of high-sensitivity cardiac troponin (hs-cTn) that were related to increased short-term and mid-term mortality, although patients were negative at PCR testing and were recovering from SARS-CoV-2 infection [34].

Our research has significant limitations, including the fact that echocardiography is not the most sensitive or specific imaging tool for assessing cardiac function and hence may have missed small anomalies affecting future clinical prognosis. However, echocardiography is easily available and is the most often used imaging tool in clinical practice for monitoring heart function. Another disadvantage is the small sample size and retrospective character of the research, resulting in restricted and missing data, necessitating narrow scale analysis.

## 5. Conclusions

The difficult care of COVID-19 patients has necessitated the development of accurate and widely accessible prognostic indicators for accurately identifying patients at increased risk of developing serious consequences. Biological parameters are quantifiable measures that may readily be used to identify individuals with COVID-19 heart failure who are at increased risk of having a poor clinical outcome. Our results imply that cardiac damage biomarkers and acute inflammatory markers should be routinely used to predict the risk of ICU admission in heart failure patients infected with SARS-CoV-2.

## Figures and Tables

**Table 1:** Background characteristics of heart failure patients stratified by SARS-CoV-2 infection status.

Characteristics *	COVID-19 (*n* = 124)	No COVID-19 (*n* = 236)	*p*-Value **
**Age, years**			0.619
18–35	5 (4.0%)	12 (5.1%)	
35–65	51 (41.1%)	107 (45.3%)	
>65	68 (54.8%)	117 (49.6%)	
Sex			0.253
Men	74 (59.7%)	126 (53.4%)	
Women	50 (40.3%)	110 (46.6%)	
**Weight, BMI (kg/m^2^)**			0.729
<25	13 (10.5%)	27 (11.4%)	
25–30	23 (18.5%)	51 (21.6%)	
≥30	88 (71.0%)	158 (66.9%)	
**Cardiovascular risk factors**			
Smoking	49 (39.5%)	92 (39.0%)	0.921
Arterial hypertension	60 (48.4%)	126 (53.4%)	0.366
Diabetes mellitus	42 (33.9%)	57 (24.2%)	0.049
Dyslipidemia	39 (31.5%)	51 (21.6%)	0.040
**Comorbid conditions**			
Cerebrovascular disease	15 (12.1%)	32 (13.6%)	0.695
Chronic kidney disease	17 (13.7%)	27 (11.4%)	0.532
COPD	13 (10.5%)	21 (8.9%)	0.624
Hematologic disorders	10 (8.1%)	16 (6.8%)	0.654
Autoimmune disease	11 (8.9%)	14 (5.9%)	0.297
Malignancy	12 (9.7%)	9 (3.8%)	0.024
**NYHA category**			0.002
I	27 (21.8%)	64 (27.2%)	
II	41 (33.1%)	92 (38.9%)	
II	36 (29.0%)	69 (29.2%)	
IV	20 (16.1%)	11 (4.7%)	
**Complete COVID-19 Vaccination**			0.006
Yes	48 (38.7%)	127 (53.8%)	
No	76 (61.3%)	109 (46.2%)	
**Oxygen supplementation**			
No supplementation	12 (9.7%)	-	
Non-invasive ventilation	74 (59.7%)	-	
Invasive ventilation	38 (30.6%)	-	
**COVID-19 severity**			
Mild	33 (26.6%)	-	
Moderate	49 (39.5%)	-	
Severe	42 (33.9%)	-	
**Disease outcomes**			
ICU admission	41 (33.1%)	-	
In-hospital mortality	28 (22.6%)	-	

* Data reported as *n* (%) unless specified differently; ** Chi-square test and Fisher’s exact; BMI—Body Mass Index; COPD—Chronic Obstructive Pulmonary Disease; ACE—Angiotensin-Converting Enzyme; ICU—Intensive Care Unit.

**Table 2:** Biological profile comparison between heart failure patients stratified by SARS-CoV-2 infection status.

Variables *	Normal Range	COVID-19 (*n* = 124)	% Outside Normality	No COVID-19 (*n* = 236)	% Outside Normality	*p*-Value **
**Complete blood count**						
RBC (millions/mm^3^)	4.35–5.65	3.46^ (1.6)	61.3%	3.52^ (1.4)	60.2%	0.836
PLT (thousands/mm^3^)	150–450	98^ (109)	62.1%	103^ (98)	54.2%	0.152
WBC (thousands/mm^3^)	4.5–11.0	13.1^ (7.5)	48.4%	4.7 (2.9)	20.3.%	<0.001
Neutrophils (thousands/mm^3^)	1.5–8.0	7.4 (4.3)	24.2%	4.9 (3.6)	10.6%	<0.001
Monocytes (thousands/mm^3^)	0.1–1.0	0.3 (0.4)	7.3%	0.6 (0.3)	9.3%	0.794
Eosinophils (units/mm^3^)	30–300	166 (91)	3.2%	163 (101)	5.1%	0.720
Lymphocytes (thousands/mm^3^)	1.0–4.8	7.0^ (6.3)	65.3%	2.6 (3.2)	36.9%	<0.001
Hemoglobin (g/dL)	13.0–17.0	12.4^ (4.3)	52.4%	12.9^ (5.1)	45.8%	0.318
Hematocrit (%)	36–48	39 (10)	18.5%	40 (12)	16.5%	0.916
MCV (fL)	80–96	87 (92)	30.6%	88 (89)	34.3%	0.644
**Liver function tests**						
Fasting glucose (mmol/L)	60–125	144^ (89)	72.6%	129^ (82)	63.9%	0.029
ALT (U/L)	7–35	59^ (44)	69.4%	45^ (37)	55.9%	0.038
AST (U/L)	10–40	42^ (32)	29.8%	36 (31)	18.2%	0.033
ALP (U/L)	40–130	104 (86)	23.4%	98 (78)	19.5%	0.482
Serum albumin (g/dL)	3.4–5.4	3.7 (1.2)	14.5%	3.8 (1.3)	13.9%	0.663
Total proteins (g/dL)	6.0–8.3	6.3 (3.4)	16.9%	6.3 (2.6)	16.1%	0.908
Total bilirubin (g/dL)	0.3–1.2	1.3^ (0.9)	32.2%	1.1 (0.7)	22.4%	0.062
GGT (U/L)	0–30	28 (19)	23.4%	26 (16)	21.6%	0.154
LDH (U/L)	140–280	241 (135)	16.9%	233 (129)	16.1%	0.891
PT (seconds)	11.0–13.5	14.4^ (7.6)	41.1%	11.3 (4.5)	23.6%	<0.001
APTT (seconds)	30–40	39 (11)	21.8%	37 (9)	17.4%	0.128
**Inflammatory markers**						
Procalcitonin (ug/L)	0–0.5 ug/L	0.7^ (0.4)	28.2%	0.5^ (0.3)	25.0%	0.194
CRP (mg/L)	0–10 mg/L	53^ (29)	71.8%	13^ (8)	23.3%	<0.001
IL-6 (pg/mL)	0–16 pg/mL	48^ (21)	62.9%	15 (7)	16.5%	<0.001
TNF-α (pg/mL)	0–29 pg/mL	43^ (17)	53.2%	22 (10)	15.7%	<0.001
IFN-γ (pg/mL)	0–3 pg/mL	3.3^ (1.5)	22.6%	2.8^ (1.3)	17.4%	0.494
ESR (mm/h)	0–22 mm/hr	66^ (28)	68.5%	25^ (13)	27.1%	<0.001
Fibrinogen (g/L)	2–4 g/L	6.1^ (2.9)	32.2%	4.1^ (1.1)	19.9%	0.003
D-dimer (ng/mL)	<250	348^ (192)	61.3%	244 (97)	19.1%	<0.001
BNP (pg/mL)	<100	398^ (206)	77.5%	262^ (146)	52.9%	<0.001
CK-MB (U/L)	5–25	33^ (14)	33.9%	26^ (8)	22.0%	0.015
LDH (U/L)	140–280	301^ (144)	38.7%	233 (129)	18.6%	<0.001
Myoglobin (nmol/L)	1.2–3.6	3.9^ (2.5)	25.0%	3.5 (1.6)	15.3%	0.023
Troponin I (ng/mL)	0–0.4	0.5^ (0.3)	16.3%	0.3^ (0.2)	11.4%	0.040
Troponin T (ng/mL)	<14	16^ (12)	30.6%	12 (8)	20.8%	0.037
**Kidney function tests**						
Creatinine (µmol/L)	0.74–1.35	1.66^ (1.69)	63.7%	1.39^ (1.51)	52.5%	0.002
BUN (mmol/L)	2.1–8.5	17^ (12)	71.8%	11^ (9)	61.0%	0.009
Urinary albumin (mg/g)	0–30	43^ (14)	62.9%	40^ (11)	58.8%	0.516
eGFR	>60	44^ (30)	69.4%	55^ (24)	54.7%	0.045
**Lipid profile**						
Total cholesterol (mg/dL)	100–200	233^ (65.8)	38.7%	226^ (58.6)	35.6%	0.292
Triglycerides	50–150	163^ (49.7)	27.4%	152^ (42.4)	22.0%	0.326
LDL-C (mg/dL)	<100	109.2^ (46.8)	22.6%	106.1^ (43.1)	21.6%	0.694
HDL-C (mg/dL)	40–60	33.0^ (16.1)	25.0%	36.4^ (15.4)	23.3%	0.657

* Data reported as median (IQR) unless specified differently; ** Mann–Whitney U-test; ^ median value outside the normal range; WBC—White Blood Cells; RBC—Red Blood Cells; AST—Aspartate Aminotransferase; ALT—Alanine Aminotransferase; ALP—Alkaline Phosphatase; eGFR—Estimated Glomerular Filtration Rate; LDH—Lactate Dehydrogenase; GGT—Gamma Glutamyl Transpeptidase; BUN—Blood Urea Nitrogen; PT—Prothrombin Time; APTT—Activated Partial Thromboplastin clotting Time; LDL—Low-Density Lipoproteins; HDL—High-Density Lipoproteins; CRP—C-reactive Protein; IL—Interleukin; TNF—Tumor Necrosis Factor; IFN—Interferon; ESR—Erythrocyte Sedimentation Rate; BNP—Brain Natriuretic Peptide; CK-MB—Creatine Kinase—Myoglobin Binding.

**Table 3:** Comparison of inflammatory markers by NYHA classification of hospitalized heart failure patients with COVID-19.

Variables *	Normal Range	NYHA I (*n* = 27)	NYHA II (*n* = 41)	NYHA III (*n* = 36)	NYHA IV (*n* = 20)	*p*-Value
Procalcitonin (ug/L)	0–0.5 ug/L	0.6 (0.3)	0.6 (0.3)	0.7 (0.3)	0.9 (0.5)	0.140
CRP (mg/L)	0–10 mg/L	51 (22)	57 (20)	54 (24)	54 (21)	0.348
IL-6 (pg/mL)	0–16 pg/mL	40 (19)	49 (21)	55 (23)	53 (19)	0.203
TNF-α (pg/mL)	0–29 pg/mL	38 (15)	44 (17)	49 (16)	43 (18)	0.417
IFN-γ (pg/mL)	0–3 pg/mL	2.7 (1.1)	2.9 (1.8)	3.3 (2.0)	3.5 (1.4)	0.264
ESR (mm/h)	0–22 mm/hr	61 (25)	72 (31)	77 (34)	76 (29)	0.094
Fibrinogen (g/L)	2–4 g/L	6.0 (2.7)	6.6 (3.1)	6.7 (3.5)	7.2 (4.0)	0.048
D-dimer (ng/mL)	<250	361 (149)	374 (160)	404 (175)	438 (192)	0.066
BNP (pg/mL)	<100	248 (93)	302 (148)	569 (291)	1170 (384)	<0.001
CK-MB (U/L)	5–25	22 (6.1)	24 (8.3)	29 (9.0)	37 (15.2)	<0.001
LDH (U/L)	140–280	208 (96)	254 (132)	291 (148)	336 (174)	0.012
Myoglobin (nmol/L)	1.2–3.6	1.6 (0.5)	1.9 (0.9)	2.8 (1.2)	3.9 (1.8)	<0.001
Troponin I (ng/mL)	0–0.4	0.1 (0.1)	0.2 (0.1)	0.4 (0.2)	0.6 (0.3)	<0.001
Troponin T (ng/mL)	<14	11 (4)	14 (6)	19 (7)	23 (11)	<0.001

* Data reported as median (interquartile range); CRP—C-reactive Protein; IL—Interleukin; TNF—Tumor Necrosis Factor; IFN—Interferon; ESR—Erythrocyte Sedimentation Rate; BNP—Brain Natriuretic Peptide; CK-MB—Creatine Kinase—Myoglobin Binding; LDH—Lactate Dehydrogenase.

**Table 4:** Comparison of cardiac markers between vaccinated and unvaccinated heart failure patients with COVID-19 at admission.

Cardiac Markers *	Vaccinated (*n* = 48)	Unvaccinated (*n* = 76)	*p*-Value **
BNP	29 (60.4%)	52 (68.4%)	0.361
CK-MB	15 (31.3%)	34 (44.7%)	0.134
LDH	18 (37.5%)	32 (42.1%)	0.610
Myoglobin	12 (25.0%)	27 (35.5%)	0.218
Troponin I	13 (27.1%)	28 (36.8%)	0.260
Troponin T	13 (27.1%)	27 (35.5%)	0.327

* Data reported as *n* (% outside normality); ** Chi-square test and Fisher’s exact; BNP—Brain Natriuretic Peptide; CK-MB—Creatine Kinase—Myoglobin Binding; LDH—Lactate Dehydrogenase.

**Table 5:** Reevaluation of patients with heart failure at 4 weeks after SARS-CoV-2 infection clearance.

Investigations	At Admission (*n* = 124)	At 4 Weeks (*n* = 96)	*p*-Value **
**Clinical profile**			0.047
NYHA I	27 (21.8%)	14 (14.6%)	
NYHA II	41 (33.1%)	23 (24.0%)	
NYHA III	36 (29.0%)	30 (31.2%)	
NYHA IV	20 (16.1%)	29 (30.2%)	
Ultrasound examination *			
LVMI	28 (22.6%)	24 (25.0%)	0.675
Pericardial effusion	23 (18.5%)	31 (32.3%)	0.018
MAPSE	49 (39.5%)	43 (44.8%)	0.431
LV-GLS	56 (45.2%)	57 (59.4%)	0.036
LV-DD	33 (26.6%)	38 (39.6%)	0.041
TAPSE	31 (25.0%)	32 (33.3%)	0.175
RVD	34 (27.4%)	38 (39.6%)	0.056
sPAP	26 (21.0%)	32 (33.3%)	0.038
TRV	25 (20.2%)	29 (30.2%)	0.085
Inflammatory markers *			
Procalcitonin	35 (28.2%)	43 (44.8%)	0.010
CRP	89 (71.8%)	62 (64.6%)	0.254
IL-6	78 (62.9%)	49 (51.0%)	0.077
TNF-α	66 (53.2%)	42 (43.8%)	0.163
IFN-γ	28 (22.6%)	13 (13.5%)	0.087
ESR	85 (68.5%)	53 (55.2%)	0.042
Fibrinogen	40 (32.2%)	26 (27.1%)	0.406
D-dimer	76 (61.3%)	41 (42.7%)	0.006
BNP	96 (77.5%)	64 (66.7%)	0.075
CK-MB	39 (31.5%)	18 (18.8%)	0.032
LDH	48 (38.7%)	32 (33.3%)	0.411
Myoglobin	31 (25.0%)	13 (13.5%)	0.035
Troponin I	45 (36.3%)	22 (22.9%)	0.032
Troponin T	51 (41.1%)	25 (26.0%)	0.019

* Data reported as *n* (% outside normality); ** Chi-square test and Fisher’s exact; LVMI—left ventricular hypertrophy; MAPSE—mitral annular plane systolic excursion; LV-GLS—left ventricular global longitudinal strain; LV-DD—Left Ventricular Diastolic Dysfunction; TAPSE—tricuspid annular plane systolic excursion; RVD—right ventricular dysfunction; sPAP—Pulmonary Artery Systolic Pressure; TRV—tricuspid regurgitation velocity; CRP—C-reactive Protein; IL—Interleukin; TNF—Tumor Necrosis Factor; IFN—Interferon; ESR—Erythrocyte Sedimentation Rate; BNP—Brain Natriuretic Peptide; CK-MB—Creatine Kinase—Myoglobin Binding; LDH—Lactate Dehydrogenase.

## Data Availability

The data presented in this study are available on request from the corresponding author.
